# Enhanced Therapeutic Efficacy of Omeprazole Nanosuspension in Ethanol-Induced Gastric Ulcer: A Focus on Oxidative Stress and Inflammatory Pathways

**DOI:** 10.3390/biom15060902

**Published:** 2025-06-19

**Authors:** Mody Albalawi, Sahar Khateeb

**Affiliations:** 1 Department of Biochemistry, Faculty of Science, University of Tabuk, Tabuk 71491, Saudi Arabia; ms_albalawi@ut.edu.sa; 2 Biochemistry Division, Department of Chemistry, Faculty of Science, Fayoum University, Fayoum 63514, Egypt

**Keywords:** omeprazole, nanosuspensions, gastric ulcer, ethanol, ROS, inflammation, bioavailability, Nrf2/PPAR-γ/SIRT1 pathway, cytokines

## Abstract

Gastric ulcer is a concerning condition that affects numerous individuals globally. Omeprazole (OMP), a well-known drug for treating stomach ulcers, has been associated with several adverse effects and limited solubility. The study aimed to create an omeprazole nanosuspension (OMP-NS) with improved solubility and bioavailability. Additionally, the study investigated the potential therapeutic effects of OMP-NS on ethanol-induced gastric injury in rats, comparing it to traditional OMP therapy to identify novel therapeutic alternatives. The characterization of the OMP-NS was assessed using DLS, TEM, XRD, FTIR, UV spectrophotometric analysis, in vitro release studies, and entrapment efficiency (EE) assays. A total of 24 male Wistar albino rats (weighing 150–200 g, aged 8–10 weeks) were randomly divided into four groups (six rats/group). Gastric injury was induced using absolute ethanol (5 mL/kg), followed by oral administration of either OMP or OMP-NS (20 mg/kg) for 7 days. Data were analyzed using one-way ANOVA accompanied by the Bonferroni post hoc test or the Kruskal–Wallis test, based on data distribution, with significance set at *p* < 0.05. The OMP-NS demonstrated a Z-average diameter of 216.1 nm, a polydispersity index of 0.2, and a zeta potential of −19.6 mV. The particles were predominantly spherical with an average size of 67.28 nm. In vitro release studies showed 97.78% release at 8 h, with an EE% of 96.97%. Ethanol-induced gastric ulcers were associated with oxidative stress, characterized by elevated levels of NADPH, ROS, MDA, and NO, while the level of SOD was reduced. It was accompanied by increased inflammatory markers HMGB1, which subsequently increased TLR-2, MyD88, NF-κBp56, NLRP3, TNF-α, IL-1β, and IL-6 levels; conversely, a significant decrease in Nrf2/PPAR-γ/SIRT1 levels was observed. In contrast, OMP-NS administration significantly reduced oxidative stress and inflammatory markers, restored SOD activity, and upregulated protective pathways Nrf2/PPAR-γ/SIRT1 more effectively than conventional OMP therapy. In conclusion, OMP-NS represents a promising therapeutic strategy with notable anti-inflammatory and anti-ulcerogenic effects in ethanol-induced gastric ulcers.

## 1. Introduction

Gastric ulcers are prevalent gastrointestinal disorders affecting around 10% of the global population [1]. They may develop as a result of excessive acid exposure, ischemia, hypoxia, nonsteroidal anti-inflammatory drug use, smoking, Helicobacter pylori infection, and alcohol intake [2]. Oxidative stress and inflammation have been recognized as the primary factors responsible for mucosal damage caused by ethanol intake. The ethanol-induced gastric ulcer model promotes oxidative stress by elevating reactive oxygen species (ROS), malondialdehyde (MDA), and inflammatory cytokines while diminishing antioxidant enzyme levels [3]. 

High mobility group box 1 (HMGB1) is a chromatin-binding factor that acts as a pro-inflammatory agent and is crucial in inflammation. HMGB1 has been identified as a contributor to stomach ulceration and the generation of inflammatory cytokines [4]. Furthermore, a previous study indicates that HMGB1 serves as a prevalent injury biomarker that activates the nucleotide-binding domain and leucine-rich repeat-containing protein 3 (NLRP3) inflammasome [5], which contains intracellular cytoplasmic multiproteins linked to inflammatory diseases. Toll-like receptor 2 (TLR-2) is crucial for the gastrointestinal mucosal immune response and may induce inflammatory reactions upon activation. Myeloid differentiation factor 88 (MyD88) serves as the primary adapter for TLR-2. Numerous studies demonstrate that the TLR-2/MyD88 signaling pathway is critical for the formation and progression of gastric ulcers [6,7]. Conversely, many mechanisms mediate their gastroprotection against oxidative damage by attenuating the inflammatory response. Nuclear factor erythroid 2-related factor 2 (Nrf2) is essential for protecting the gastrointestinal tract from oxidative damage [8]. Peroxisome proliferator-activated receptor gamma (PPAR-γ) is a further factor that supports gastroprotection against oxidative stress by diminishing the inflammatory response [9]. Furthermore, Sirtuin 1 (SIRT1) is an NAD^+^-dependent histone deacetylase involved in various physiological and pathological signaling pathways [10]; it mitigates inflammation by regulating the nuclear factor kappa B (NF-κB) signaling pathway [11].

Omeprazole (OMP) is commonly used to treat gastric ulcers; however, it has been linked with numerous side effects [12]. OMP is usually available as enteric-coated capsules to prevent degradation by stomach acid [13]; however, oral administration of these enteric-coated solids may pose challenges for pediatric patients, individuals unable to swallow capsules, or patients requiring less than a full capsule dose. Furthermore, administration via nasogastric tubes is problematic, as the capsules can clog the tube even when prepared by specialized pharmacists. Moreover, OMP is a poorly soluble [14], chemically unstable drug with a high degradation rate in aqueous environments. To address these limitations, nanomaterials have been developed to enhance their solubility and bioavailability.

Nanosuspensions (NS) are a promising method to improve the bioavailability of hydrophobic drugs [15]. NS are characterized as distinct liquid submicron colloidal dispersions of nanoscale pure drug particles, stabilized by polymers or surfactants, with a particle size ranging from 1 to 1000 nm [16]. These nanosystems improve the solubility of poorly water-soluble drugs due to their small size and large surface area [15,17,18]. Moreover, they can modify the pharmacokinetics of the medicine, thereby improving its efficacy and safety [19], by enhancing drug loading, improving dose-bioavailability correlation, reducing toxicity and side effects, and fostering patient adherence by decreasing the number of oral units required for administration [20]. The objective of this study was to create and characterize an omeprazole nanosuspension (OMP-NS) with enhanced solubility and bioavailability and to assess its therapeutic effects on ethanol-induced gastric injury in rats, comparing its efficacy to traditional OMP therapy to identify potential alternative treatments.

## 2. Materials and Methods

### 2.1. Materials

OMP powder was procured from Sigma (Louis, MO, USA). Poloxamer 188 (USP/NF, CAS number 9003-11-6) was procured from BASF Corporation (Florham Park, NJ, USA), while polyvinyl alcohol (PVA) was sourced from EMPROVE^®^ ESSENTIAL (Darmstadt, Germany). The other compounds utilized in the investigation were of analytical grade.

### 2.2. Preparation of OMP-NS

OMP-NS was formulated using the methodology of Gupta et al. [21]. Pure OMP and poloxamer 188 were dissolved in 5 mL of methanol at room temperature to create a homogeneous organic solution. The organic solution was subsequently injected dropwise using a syringe into an aqueous phase (10 mL) containing stabilizers (PVA) under high-speed mechanical agitation at 1000 rpm to achieve the desired nano-dispersion. The prepared OMP-NS was subsequently agitated magnetically at 500 rpm at ambient temperatures for 12 h to facilitate the evaporation of the organic solvent. The complete evaporation of methanol was ascertained using a spectrophotometric approach. The volume was subsequently modified by adding triple distilled water to compensate for losses while maintaining other parameters constant. 

### 2.3. Characterization of OMP-NS

The particle size and size distribution of the created OMP-NS were evaluated in relation to average volume diameters and polydispersity index (PDI) utilizing a particle size analyzer, Dynamic Light Scattering (DLS) (Zetasizer Nano ZN, Malvern Panalytical Ltd., Malvern, UK). Transmission electron microscopy (TEM) was performed using a Thermo Scientific Talos F200i (S)TEM (Thermo Fisher Scientific, USA), operating at 20–200 kV. The X-ray diffraction (XRD) pattern of the created OMP-NS was obtained using a PANalytical X’Pert PRO diffractometer (PANalytical B.V., Almelo, The Netherlands). Data analysis was conducted using X’Pert HighScore. Fourier transform infrared (FTIR) spectroscopy was used to obtain the spectra of OMP and OMP-NS. The existence of functional groups was analyzed using FTIR (Nexus 670, Nicollet, Madison, WI, USA) in the mid-infrared range of 400–4000 cm^−1^. The maximum absorbance wavelength (λ_max) of OMP and OMP-NS was determined by analyzing a 100 μg/mL solution in the wavelength range of 280–400 nm using an E-2100UV spectrophotometer (Peak Instruments Inc., Houston, TX, USA). 

### 2.4. Preparation of Calibration Curve

**Figure 1:**
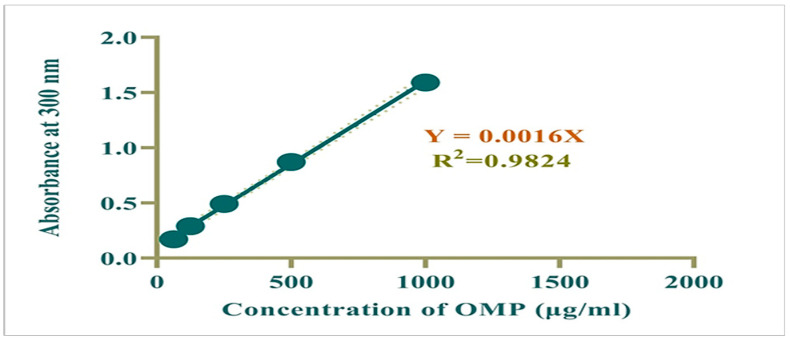
Calibration curve for OMP.

Standard working solutions of OMP (62.5–500 μg/mL) were produced through serial dilution of a 1000 μg/mL stock solution using methanol. Absorbance of OMP was measured at 300 nm, a calibration curve was plotted, and the regression equation was determined. The correlation coefficient (R^2^) for this calibration curve was calculated to be 0.982, indicating a strong linear relationship between absorbance and concentration (Figure 1).

### 2.5. In Vitro Release Study and Entrapment Effectiveness (EE%)

The drug release from the OMP-NS was assessed using a diffusion method with a dialysis bag (12,000 MW) in phosphate-buffered saline (PBS) at pH 7.4. The formulation was placed in a dialysis bag and then immersed in 100 mL of PBS. The entire apparatus was maintained at 37 ± 1 °C and stirred at 500 rpm. At designated time intervals (1, 2, 3, 4, 5, 6, 7, and 8 h), 2 mL samples were extracted and replaced with new buffer. The samples were analyzed using an E-2100UV spectrophotometer at 300 nm. 

The drug EE (%) of the formulation was determined as a percentage according to the formula provided by Kathle et al. [22].

$$EE\left(\%\right)=\frac{W\left(total\;drug\right)-W\left(free\;drug\right)}{W(total\;drug)}\times 100\%$$

### 2.6. Gastric Ulcer Induction and Experimental Grouping

Twenty-four healthy male Wistar rats, aged 8–10 weeks and weighing between 150 and 200 g, were used. All animals had a 24 h fast prior to ethanol administration, with free access to water. Gastric ulcers were induced by a single oral dose of absolute ethanol (5 mL/kg) [23,24] through intragastric gavage. The experimental subjects were randomly allocated into four groups (*n* = 6). The control group consisted of rats that were administered normal saline (5 mL/kg) orally for 7 days. The ulcer group comprised rats that received absolute ethanol (5 mL/kg) on day 1, followed by oral administration of normal saline (5 mL/kg) for 7 days. In the ulcer + OMP group, rats were administered absolute ethanol (5 mL/kg), followed by oral OMP (20 mg/kg) for 7 days. In the ulcer + OMP-NS group, rats received absolute ethanol (5 mL/kg), followed by oral OMP-NS (20 mg/kg) for 7 days. 

### 2.7. Sample Collection

At the end of the 7-day study period, rats were euthanized following anesthesia with ketamine (70 mg/kg, i.p.) and xylazine (10 mg/kg, i.p.) [25]. The remaining number of rats in each group was as follows: control (*n* = 6), ulcer (*n* = 4), OMP (*n* = 5), and OMP-NS (*n* = 6). Blood specimens were obtained via cardiac puncture into a serum tube and centrifuged at 3000 rpm for 5 min at 4 °C to obtain serum aliquots, which were subsequently kept at −20 °C until analysis. Following the experimental protocol, glandular sections of stomach tissue were carefully excised and rinsed with ice-cold normal saline to remove any residual debris [26]. The stomachs were then incised along the greater curvature, allowing for the collection of stomach secretions. These secretions were centrifuged to separate the supernatant gastric fluid, which was subsequently analyzed to determine its pH level [27]. Stomach tissues were partitioned into two parts: one for histological evaluation, preserved in 10% formalin, and the other for the preparation of gastric homogenate for biochemical analysis.

### 2.8. Determination of Gastric pH, Assessment of Gastric Mucosal Damage, and Ulcer Index (U.I.)

The pH of the gastric juice was assessed using a tabletop pH meter (HANNA, HI 110, Smithfield, RI, USA). The gastric cavity of each rat was washed with cold saline and gently dried with absorbent filter sheets. Subsequently, digital imaging of the stomachs was conducted, facilitating the evaluation of potential damage to the mucosal lining. A macroscopic assessment of the stomach was performed to detect any hemorrhagic lesions on the glandular mucosa [28,29]. The images were analyzed using ImageJ2 software (version version 2.16.0; Wayne Rasband, Kensington, MD, USA). The program was employed to quantify the areas of ulcerations, and thereafter, the ulceration ratio was calculated. The degree of ulceration in the stomach mucosa was measured to assess ulcer severity [30]. U.I. for each rat was calculated according to the methodology delineated by Szabo and Hollander [31], using the subsequent formula:

$$U.I.(\%)=\frac{Ulcerated\;area}{Total\;stomach\;area}\times 100$$

The curative rate (CR%) against ulceration was calculated using the following formula:

$$CR(\%)=\frac{U.I.\;in\;ulcer\;group-U.I.\;in\;test\;group}{U.I.\;in\;ulcer\;group}\times 100$$

### 2.9. Biochemical Analysis

#### 2.9.1. Assessment of Hepatic and Renal Biomarkers

The activity of alanine aminotransferase (ALT), aspartate aminotransferase (AST), and alkaline phosphatase (ALP) was assessed by diagnostic kits (Spectrum Diagnosis, Cairo, Egypt), following the manufacturer’s instructions. Urea (Abcam, Catalog No. ab83362, Cambridge, UK), creatinine (Bio-diagnostic, Cairo, Egypt), and uric acid (Cat. No. MA-UA; RayBiotech Inc., Peachtree Corners, GA, USA) concentrations were quantified in serum in accordance with the manufacturer’s guidelines.

#### 2.9.2. Assessment of Oxidative Stress Biomarkers

In accordance with the manufacturer’s instructions, a spectrophotometric kit (Bio-diagnostic, Cairo, Egypt) was employed to quantify MDA, nitric oxide (NO), and superoxide dismutase (SOD) activity in homogenates.

#### 2.9.3. Enzyme-Linked Immunosorbent Assay (ELISA)

Cortisol levels were assessed utilizing the Rat Cortisol ELISA Kit (MyBioSource, San Diego, CA, USA) (Catalog No. MBS265522), whereas gastrin levels were determined using the Rat Gastrin I Enzyme Immunoassay Kit (Catalog No. MBS494300) in gastric juice, adhering to the manufacturer’s guidelines. ROS levels were measured using the Rat ROS ELISA Kit (Cat. No. MBS039665). Nicotinamide adenine dinucleotide phosphate (NADPH) levels were quantified using the Rat NADPH ELISA Kit (Cat. No. MBS2506694). HMGB-1 was assessed using the ELISA Kit (Catalog No. MBS703437). NLRP3 levels were determined using the ELISA Kit (Cat. No. MBS7255410). NF-κB was quantified using the ELISA Kit (Cat. No. MBS453975). Interleukin-6 (IL-6), IL-1β, and tumor necrosis factor alpha (TNF-α) were measured using the ELISA Kit (cat. No. SEA079Ra, SEA563Ra, and SEA133Ra, respectively). TLR2 and MyD88 were measured using the ELISA Kit (Cat. No. MBS2022789 and MBS2703631, respectively), following the manufacturer’s instructions for all kits in homogenates.

#### 2.9.4. RNA Extraction and Quantitative RT-PCR Analysis

Total RNA was isolated from tissue lysate using Direct-zol RNA Miniprep Plus (Cat# R2072, ZYMO RESEARCH CORP., Tustin, CA, USA) and subsequently assessed for quantity and quality with a Beckman dual spectrophotometer (Beckman Coulter, Brea, CA, USA). Reverse transcription into cDNA was conducted using the Invitrogen™ SuperScript™ IV One-Step RT-PCR System from Thermo Fisher Scientific, Waltham, MA, USA, in accordance with the manufacturer’s guidelines. The expression levels of target genes in each sample were evaluated using the SuperScript IV One-Step RT-PCR kit (Cat# 12594100, Thermo Fisher Scientific, Waltham, MA, USA). Gene expression was assessed using threshold cycle values and normalized against the housekeeping gene, GAPDH. The qRT-PCR primers for PPAR-γ, SIRT-1, and Nrf2 are listed in Table 1.

### 2.10. Histopathological Analysis

Gastric specimens were processed by dehydration in ascending concentrations of ethanol, cleared in xylene, embedded in paraffin wax, and sectioned at 5 µm thickness. Sections were stained with hematoxylin and eosin (H&E) and examined under a light digital microscope (Olympus XC30, Tokyo, Japan) [32]. Histopathological changes in the gastric tissue were assessed semi-quantitatively in five randomly selected high-power fields (20× magnification) per section. The following parameters were evaluated: epithelial loss, necrosis, edema, and inflammatory cell infiltration. Each parameter was scored based on severity using a 4-point scale: 0 = normal, 1 = mild, 2 = moderate, and 3 = severe alterations. This scoring system was adapted from previously published histological scoring methods for gastric injury [33,34] with minor modifications.

### 2.11. Statistical Analysis

The statistical analysis for the current investigation was conducted using GraphPad Prism Software version 9.5.1 (GraphPad Software Inc., San Diego, CA, USA). Experimental data obtained from the animal models were expressed as mean ± standard deviation (SD). One-way analysis of variance (ANOVA) was utilized to assess differences among groups, followed by the Bonferroni test for multiple comparisons to evaluate significance at a 95% confidence level. Differences were considered statistically significant when *p* < 0.05. To assess the distribution of data for the histopathological scoring, the Shapiro–Wilk test for normality was applied. Since the scoring data were not normally distributed, nonparametric statistical analysis using the Kruskal–Wallis test was performed for this parameter only. In contrast, all other quantitative measurements followed a normal distribution and were analyzed using parametric statistical methods.

The drug EE% was analyzed using a one-sample Wilcoxon signed-rank test to determine if the observed efficiency significantly differed from a predetermined theoretical value. Sample size determination was conducted using G Power 3.1.9.4 software (Heinrich Heine University Düsseldorf, Düsseldorf, Germany) according to Faul et al. [35], ensuring adequate power to detect statistically significant differences among groups. A total of 24 adult male Wistar rats (6 rats/group) were included in the study based on a significance level (α) of 0.05 and testing power (1-β) of 0.8 for four groups, taking into account preliminary results and calculating an effect size of 1.02.

## 3. Results

### 3.1. Particle Size, Zeta Potential, and PDI Analysis

**Figure 2:**
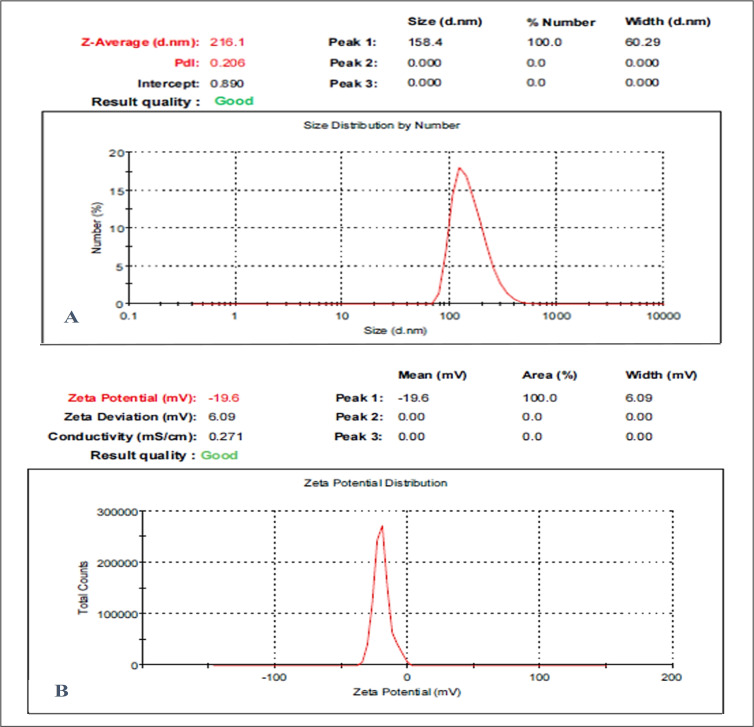
Particle size and zeta potential analysis of the OMP-NS formulation. (**A**) Size distribution of the OMP-NS showed a peak at 158.4 nm, with a Z-average size of 216.1 nm and a polydispersity index (PDI) of 0.206. The particle size ranged from approximately 100 to 300 nm; (**B**) zeta potential measurement of OMP-NS indicated a value of −19.6 mV.

The effectiveness and stability of the suspended nanoparticulate were assessed using DLS. The OMP-NS had a Z-average diameter of 216.1 nm according to the DLS analysis, and the PDI was found to be 0.2, reflecting a relatively low level of variability in particle size distribution and confirming the stability of the formulation. The OMP-NS’s zeta potential was measured at −19.6 mV, as displayed in Figure 2. This negative zeta potential suggests adequate electrostatic stabilization, which is crucial for preventing agglomeration and maintaining the NS’s stability over time. The particle size distribution was narrow, suggesting uniformity in the formulation, which is essential for consistent therapeutic performance.

### 3.2. Structural Characterization of OMP-NS Using TEM Analysis

**Figure 3:**
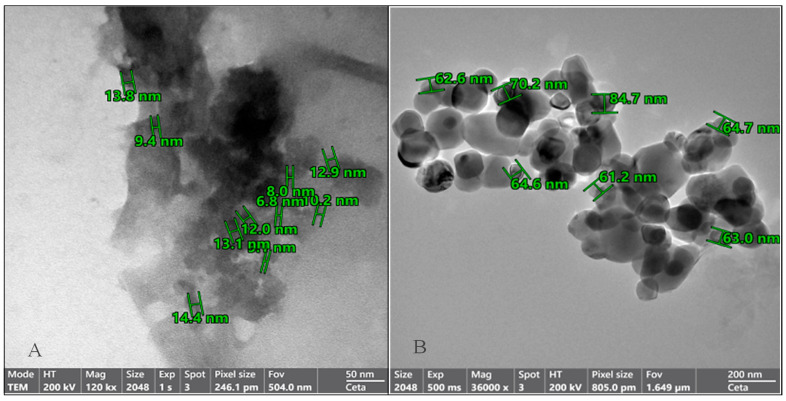
Transmission electron microscopy (TEM) images of the OMP-NS formulation. (**A**) The particle sizes of the OMP-NS ranged from 6.8 to 14.4 nm (scale bar: 50 nm). (**B**) The particle sizes of the OMP-NS ranged from 61.2 to 84.7 nm (scale bar: 200 nm).

The morphology and particle size distribution of the OMP-NS were evaluated using TEM. The TEM images in Figure 3 revealed that the OMP-NS predominantly consisted of well-defined, spherical particles, indicating successful formulation and stabilization. The mean particle size of the OMP-NS was determined to be 67.28 nm. This reduction is critical for enhancing the solubility and bioavailability of OMP.

### 3.3. XRD Analysis of OMP and OMP-NS

**Figure 4:**
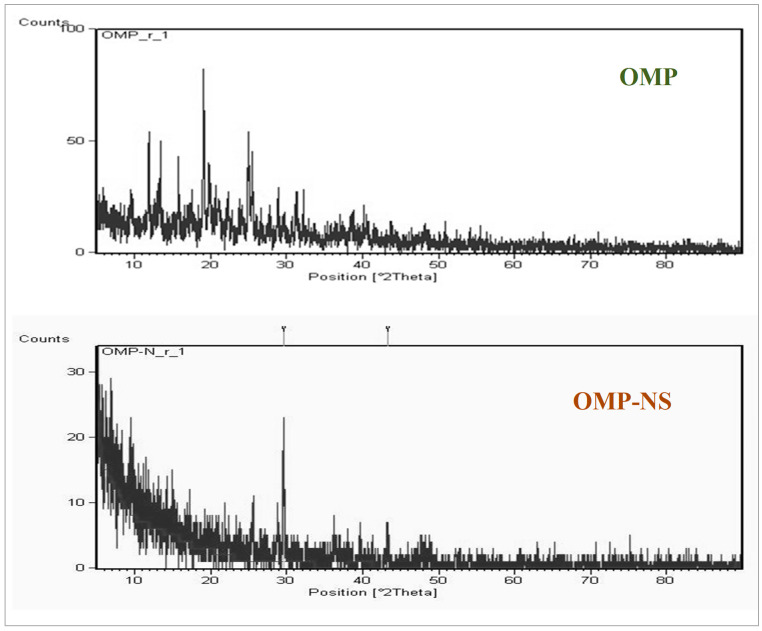
X-ray diffraction (XRD) patterns of OMP and OMP-NS.

XRD examination was conducted to assess the crystallinity and structural properties of OMP and OMP-NS. The XRD spectrum of OMP exhibited several distinct peaks. Notable peaks were observed at 11.9°, 13.3°, 15.7°, 19.06°, 19.8°, and 24.9° (2θ) (Figure 4), which correspond to specific d-spacing values characteristic of the crystalline structure of OMP. The intensity of these peaks reflects a high degree of crystallinity, confirming that the raw OMP retains its solid-state stability. In contrast, the XRD pattern of the OMP-NS showed a notable reduction in peak intensity and a decrease in the number of observable peaks compared to the raw OMP. The most prominent peaks were observed at 29.5° and 43.25° (2θ), indicating a shift in the crystalline structure and a reduction in crystallinity due to the formulation process. The diminished peak intensity suggests that a portion of the OMP has transitioned towards an amorphous state, which is consistent with the intended enhancement of solubility and bioavailability in the nanosuspension formulation.

### 3.4. FTIR Spectrum Analysis of OMP and OMP-NS Formulation 

**Figure 5:**
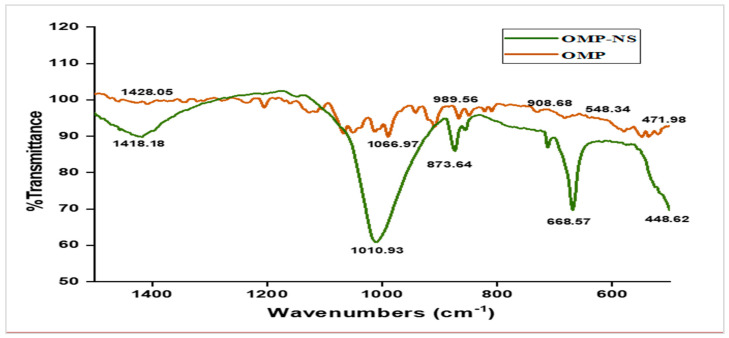
FTIR spectrum analysis of OMP and OMP-NS formulation.

The FTIR analysis was conducted to assess the molecular interactions and functional groups present in both OMP and OMP-NS. The spectrum presented in Figure 5 covers the wavenumber range of 400 to 1400 cm^−1^, with no peaks observed between 1600 and 4000 cm^−1^. Table 2 summarizes the observed frequencies for the OMP and OMP-NS. The peak at 1428.05 cm^−1^ in the FTIR spectrum of OMP shifted to 1418.18 cm^−1^ in OMP-NS, indicating a change in the electronic environment of the functional groups, likely due to interactions with stabilizers or other components in the NS. The peak at 1066.97 cm^−1^ for OMP shifted to 1010.93 cm^−1^ in OMP-NS, which may suggest alterations in bonding or molecular structure that could influence solubility and stability. A notable increase in intensity and a shift to a new peak at 668.57 cm^−1^ for OMP-NS compared to 548.34 cm^−1^ in OMP indicate possible new interactions or complex formation within the NS. The shift from 471.98 cm^−1^ in OMP to 448.62 cm^−1^ in OMP-NS further supports the notion of structural changes that may arise from the formulation process. These spectral changes indicate that the NS formulation may enhance the solubility and stability of OMP, which could lead to improved bioavailability and therapeutic efficacy.

### 3.5. UV Spectra of OMP and OMP-NS

**Figure 6:**
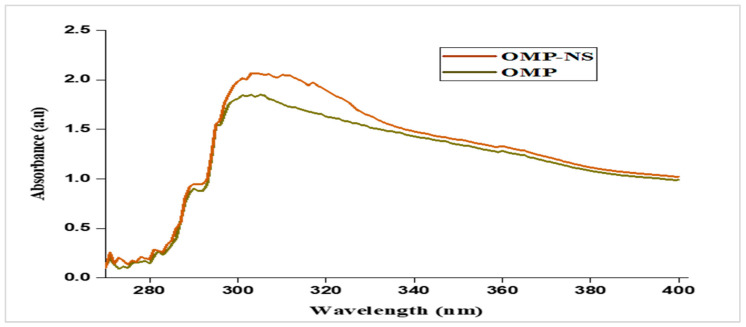
UV spectra of OMP and OMP-NS.

The UV spectrum of OMP exhibited significant absorbance peaks at approximately 300 nm (Figure 6). These peaks correspond to the electronic transitions of the OMP. The absorbance ratio at these wavelengths was calculated, showing a consistent ratio indicative of the stability and purity of the OMP sample. The UV spectrum for the OMP-NS displayed a shift in the absorbance peaks compared to the raw OMP. The primary absorbance peak was observed at 301 nm, indicating a slight alteration in the electronic environment due to the NS formulation. An increase in absorbance intensity signifies a greater quantity of nanoparticles in the solution, suggesting effective dispersion and stability of the formulation.

### 3.6. In Vitro Release Profile and EE% of OMP-NS 

**Figure 7:**
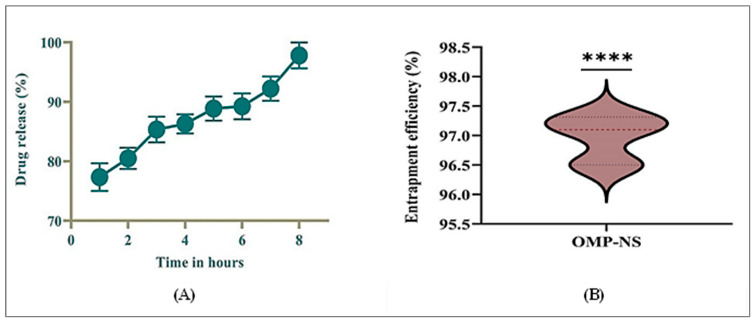
(**A**) In vitro release profile of OMP-NS and (**B**) Entrapment efficiency (EE%) of OMP-NS. **** indicates statistical significance at *p* < 0.0001.

The in vitro drug release profile of OMP-NS was assessed for a duration of 8 h. Figure 7A summarizes the percentage of drugs released at each time point. At the end of the first hour, the cumulative drug release was 77.33%, indicating a rapid initial release characteristic of the NS formulation. The release continued to increase steadily, reaching 80.49% by hour two and 85.35% by hour three, demonstrating a consistent dissolution rate during the initial hours. By the fourth hour, the cumulative release was 86.28%, and this trend continued, with values reaching 88.85% and 89.24% at hours five and six, respectively. By the end of the study period (hour eight), nearly complete drug release was achieved, with a cumulative release of 97.78%. The results indicated that OMP-NS exhibited a rapid and sustained drug release profile over 8 h, with significant amounts of the drug released within the first few hours. The near-complete release by hour eight suggested that the NS formulation effectively enhanced the solubility and bioavailability of OMP.

The EE% of OMP-NS was evaluated to determine the effectiveness of the formulation in encapsulating the drug. The results in Figure 7B demonstrated that the OMP-NS achieved a high EE% of 96.97%, with minimal variability, confirming the formulation’s potential for effective drug delivery. This high EE% is promising for enhancing the bioavailability and therapeutic efficacy of OMP in clinical applications.

### 3.7. The Macroscopic Examination of the Gastric Mucosa

**Figure 8:**
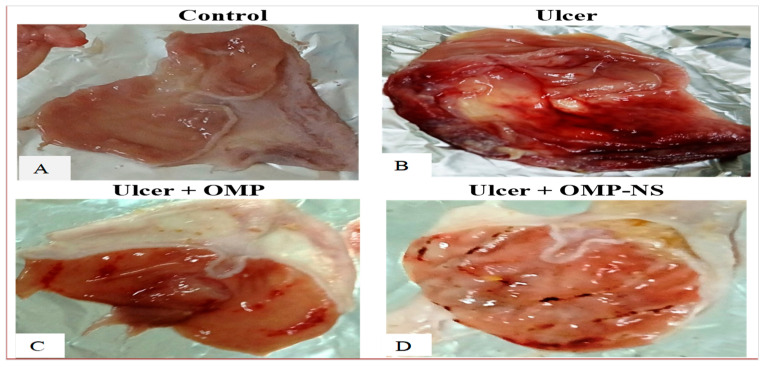
The impact of OMP and OMP-NS on the macroscopic appearance of gastric mucosa in ethanol-induced gastric damage in rats. (**A**) Control group, (**B**) ulcer group, (**C**) ulcer + OMP group, and (**D**) ulcer + OMP-NS group.

The macroscopic examination of the gastric mucosa was conducted to assess the effects of various treatments on ethanol-induced gastric mucosal damage in rats. The gastric mucosa in the control group (Figure 8A) exhibited a normal appearance with intact tissue architecture, showing no signs of lesions or hemorrhage. The ulcer group (Figure 8B) displayed extensive and severe hemorrhagic lesions on the gastric mucosa. The mucosal surface was characterized by multiple ulcerations and significant inflammation, indicating severe gastric damage. Rats that received OMP (Figure 8C) showed a marked improvement in the macroscopic appearance of the gastric mucosa compared to the ulcer group. The presence of hemorrhagic lesions was significantly reduced, and only mild inflammation was observed, indicating effective treatment. Similarly, rats treated with OMP-NS exhibited a notable reduction in gastric lesions compared to the ulcer group (Figure 8D). The mucosal surface showed minimal signs of hemorrhage and inflammation. 

### 3.8. Impact of Treatment on Body Weight, Stomach Weight, Stomach Coefficient, Gastric pH, U.I., and CR in Ethanol-Induced Gastric Ulcer

In the ulcer group, the stomach weight was significantly higher than in the control group (*p* < 0.0001), reflecting the impact of ethanol-induced damage on gastric tissue. In contrast, both the ulcer + OMP group and the ulcer + OMP-NS group exhibited significantly lower stomach weights compared to the ulcer group (Table 3), suggesting that these treatments effectively mitigated gastric injury. Similarly, the stomach coefficient was significantly elevated in the ulcer group compared to the control group. Both treatment groups demonstrated reductions in stomach coefficient values, with the ulcer + OMP-NS group being comparable to the control. 

The ulcer group exhibited markedly lower gastric pH levels than the control group, indicating an acidic environment associated with mucosal damage. Notably, the ulcer + OMP-NS group showed a significant increase in gastric pH, reflecting a more favorable gastric environment conducive to healing. Moreover, the U.I. was significantly elevated in the ulcer group, highlighting the severity of mucosal damage. Both treatment groups exhibited significant reductions in U.I. (Table 3). Furthermore, the CR for both treatment groups was significantly improved as compared to the ulcer group. Finally, a direct comparison between the ulcer + OMP and ulcer + OMP-NS groups revealed that the latter induced significantly greater improvements in final body weight (*p* < 0.01), stomach coefficient (*p* < 0.01), gastric pH (*p* < 0.001), U.I. (*p* < 0.001), and CR (*p* < 0.0001). The findings indicated that the OMP-NS formulation was more effective in reducing ulcer severity compared to OMP therapy.

### 3.9. Impact of OMP and OMP-NS on Biochemical Markers of Liver and Kidney Function 

The findings revealed substantial changes in enzyme levels related to liver function. ALT, AST, and ALP levels were markedly higher in the ulcer group versus the control group, indicating increases of approximately 174%, 152%, and 325% for ALT, AST, and ALP, respectively (Table 4). In contrast, both treatment groups, ulcer + OMP and ulcer + OMP-NS, demonstrated significant reductions in ALT levels, indicating improved liver function and a decrease of 24.5% and 47.5%, respectively, compared to the ulcer group. The ulcer + OMP group showed a reduction in AST levels, while the ulcer + OMP-NS group exhibited a further decrease, indicating protective effects of the treatments on liver integrity with reductions of 28.7% and 46.8%, respectively. Furthermore, both treatment groups exhibited substantial decreases in ALP levels, with the ulcer + OMP-NS group displaying values indicative of a restoration of normal liver function. 

Concerning renal function, creatinine levels were markedly elevated in the ulcer group. Conversely, both treatment groups exhibited substantial reductions, with ulcer + OMP and ulcer + OMP-NS indicating enhanced renal function following treatment, with reductions of 44.3% and 62.3%, respectively. Moreover, urea concentration was significantly higher in the ulcer group relative to the control group, indicating an increase of about 436%. Both treatment groups demonstrated significant reductions in urea levels. Uric acid levels were significantly greater in the ulcer group (5.48 mg/dL) in contrast to the control group (0.50 mg/dL), indicating a rise of approximately 996%. Both treatment groups exhibited substantial decreases, thereby improving renal protection (Table 4). Finally, compared to the OMP treatment, the OMP-NS formulation exhibited significantly greater reductions in ALT (*p* < 0.0001), AST (*p* < 0.0001), ALP (*p* < 0.01), creatinine (*p* < 0.01), urea (*p* < 0.0001), and uric acid levels (*p* < 0.001), indicating enhanced hepatic and renal protective effects compared to the conventional treatment.

### 3.10. Impact of Ulceration and Treatment on Cortisol and Gastrin Levels

**Figure 9:**
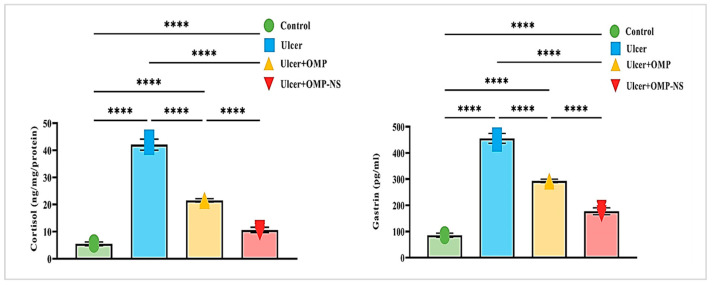
Changes in cortisol and gastrin levels across experimental groups: Data were expressed as mean ± SD. ****: significant at *p* < 0.0001 level.

The investigation of cortisol and gastrin levels across different experimental groups revealed significant hormonal changes associated with ulceration and treatment interventions. Cortisol and gastrin levels in the ulcer group exhibited a remarkable increase of approximately 671% and 431%, respectively, compared to the control group. Treatment with OMP led to a 49% decrease in cortisol levels and a 36% reduction in gastrin levels in the ulcer group, whereas OMP-NS further lowered cortisol levels by roughly 75% and gastrin levels by around 61% (Figure 9). In addition, a direct comparison between the ulcer + OMP and ulcer + OMP-NS groups revealed that the OMP-NS group exhibited significantly lower cortisol and gastrin levels than the OMP group (*p* < 0.0001), indicating a superior efficacy in modulating stress and gastric hormone responses.

### 3.11. Impact of OMP and OMP-NS on Oxidative Stress Markers 

Compared to the control group, the ulcer group exhibited a marked decrease in SOD levels by approximately 84%, alongside a substantial increase in ROS levels of around 471%, an increase in NO levels by approximately 493%, a significant elevation in MDA levels by about 496%, and a notable rise in NADPH levels of nearly 600%. Moreover, OMP treatment contributed to a 246% increase in SOD levels, a significant reduction of ROS levels by approximately 40%, a 41% reduction in NO levels, a 38% decrease in MDA levels, and a decrease in NADPH levels by about 34% relative to the ulcer group. 

**Figure 10:**
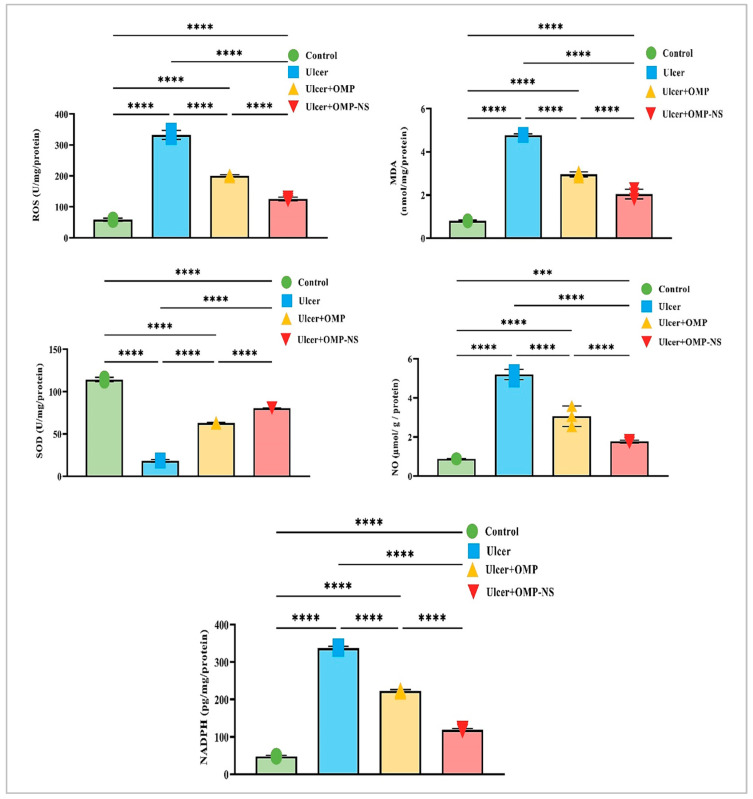
Changes in ROS, MDA, SOD, NO, NADPH levels across experimental groups. Data were expressed as mean ± SD. ***: significant at *p* < 0.001 level; and ****: significant at *p* < 0.0001 level.

Similarly, the OMP-NS treatment resulted in substantial enhancements, with SOD levels rising by around 340%, a significant decrease in ROS levels by approximately 62%, a marked decrease in NO levels by about 66%, MDA levels diminishing by approximately 57%, and NADPH levels decreasing by about 65% in comparison to the ulcer group. In addition, OMP-NS significantly elevated SOD levels and more efficiently diminished NO and MDA relative to OMP, highlighting its efficacy in reducing NO and lipid peroxidation (Figure 10).

### 3.12. Therapeutic Modulation of Inflammatory Pathways by OMP and OMP-NS in Gastric Ulceration: Focus on HMGB1/NLRP3/NF-κB, TLR-2/MyD88, and Cytokine Responses

**Figure 11:**
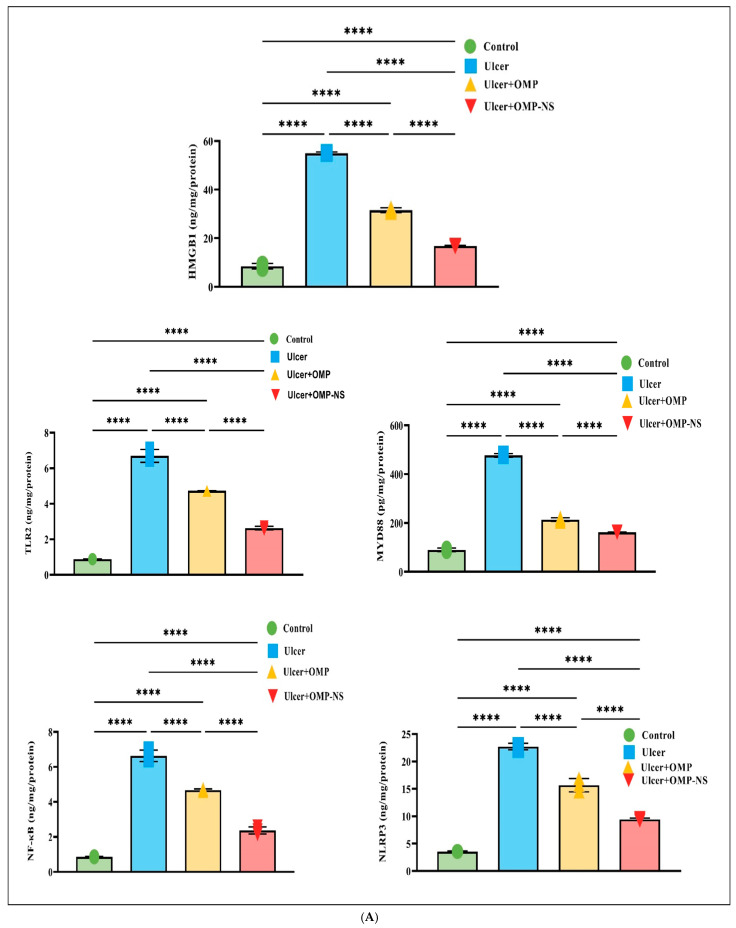

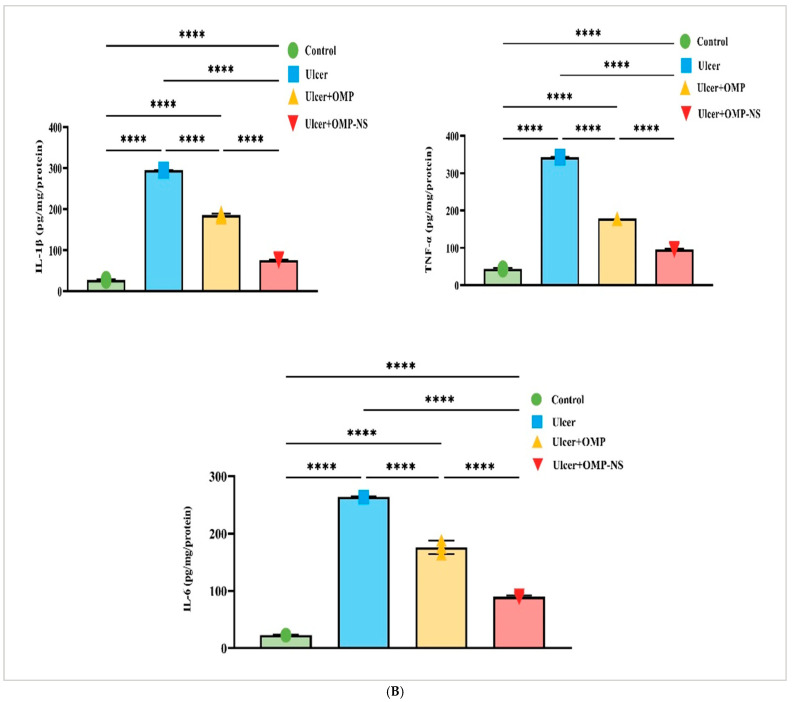
(**A**) Effects of OMP and OMP-NS on the HMGB1/NLRP3/NF-κB and TLR-2/MyD88 signaling pathway in gastric ulceration. Data were expressed as mean ± SD. ****: significant at *p* < 0.0001 level. (**B**) The levels of pro-inflammatory cytokines—IL-1β, IL-6, and TNF-α—across experimental groups. Data were expressed as mean ± SD. ****: significant at *p* < 0.0001 level.

The investigation into the HMGB1/NLRP3/NF-κB signaling pathway revealed significant alterations across the experimental groups (Figure 11A), highlighting the inflammatory response associated with gastric ulceration and the therapeutic potential of OMP and OMP-NS. In the ulcer group, HMGB1 levels were significantly increased relative to the control group, indicating its function as a critical pro-inflammatory mediator. Treatment with OMP or OMP-NS resulted in significant reductions in HMGB1 levels relative to the ulcer group, demonstrating their effectiveness in mitigating the inflammatory response. Additionally, NLRP3 levels were markedly higher in the ulcer group, indicating enhanced inflammasome activation closely associated with increased HMGB1 levels. Both treatments significantly reduced NLRP3 levels compared to the ulcer group, indicating effective modulation of inflammasome activity and a decrease in inflammation. Moreover, NF-κB levels were significantly elevated in the ulcer group relative to controls, reflecting enhanced transcriptional activity associated with inflammation. Treatment with OMP resulted in a substantial decrease in NF-κB levels, whereas OMP-NS treatment produced an even more marked reduction.

The examination of TLR-2 and MyD88 levels among experimental groups has highlighted the pathway’s importance in illustrating the inflammatory response linked to stomach ulcers. In the ulcer group, MyD88 levels were elevated, signifying an exacerbated inflammatory condition, while the notable rise in TLR-2 levels further emphasizes the intensified inflammatory response. Moreover, treatment with OMP or OMP-NS has exhibited significant ability to regulate the TLR-2/MyD88 pathway. Both treatments markedly reduced TLR-2/MyD88 levels, demonstrating their efficacy in alleviating inflammation via the TLR-2/MyD88 pathway (Figure 11A). 

The assessment of pro-inflammatory cytokines—IL-1β, IL-6, and TNF-α—among the experimental groups demonstrated considerable variations, as depicted in Figure 11B. In the ulcer group, levels of IL-1β, IL-6, and TNF-α were significantly elevated compared with the control group, indicating an intensified inflammatory condition. Treatment with OMP resulted in a significant reduction in IL-1β, IL-6, and TNF-α levels, whereas OMP-NS treatment produced an even more substantial decrease in these cytokines. This finding reinforces the ability of both OMP and OMP-NS to attenuate the levels of pro-inflammatory cytokines—IL-1β, IL-6, and TNF-α—associated with gastric ulceration. 

A direct comparison between the ulcer + OMP and ulcer + OMP-NS groups revealed that the OMP-NS formulation significantly decreased levels of HMGB1, NLRP3, NF-κB, TLR-2, MyD88, IL-1β, IL-6, and TNF-α (Figure 11). The enhanced effectiveness of OMP-NS suggests its promising therapeutic potential for modulating inflammatory responses and facilitating healing in gastric ulcers.

### 3.13. Impact of OMP and OMP-NS on Nrf2/PPAR-γ/SIRT-1 Pathways in Gastric Ulceration

**Figure 12:**
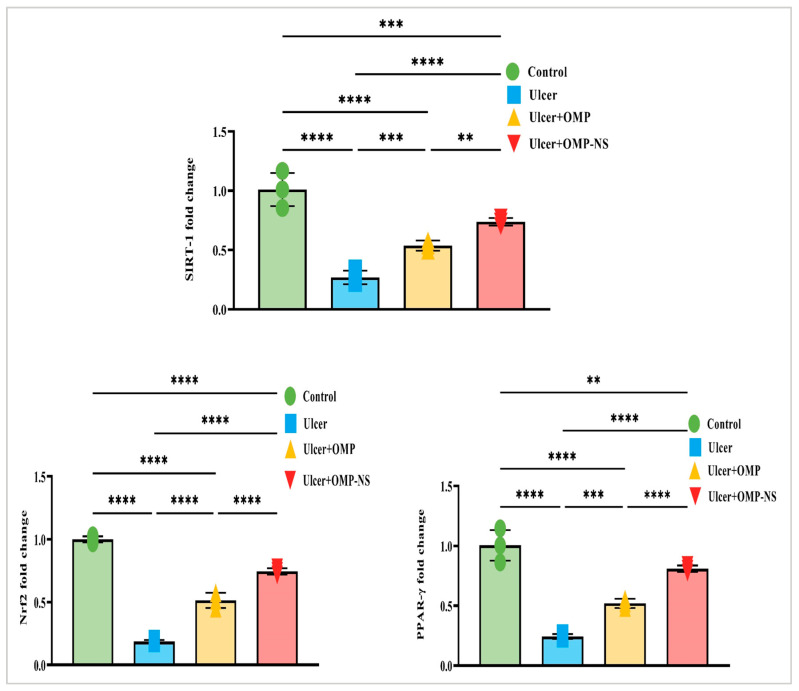
Interconnected regulation of Nrf2/PPAR-γ/SIRT-1 in gastric ulceration and treatment response. Data were expressed as mean ± SD. **: significant at *p* < 0.01 level; ***: significant at *p* < 0.001 level; and ****: significant at *p* < 0.0001 level.

Nrf2, a crucial transcription factor regulating antioxidant responses, was significantly downregulated in the ulcer group relative to controls, signifying a reduction in antioxidant defense mechanisms. Treatment with OMP or OMP-NS resulted in differing levels of Nrf2 restoration. Additionally, PPAR-γ, a nuclear receptor involved in anti-inflammatory and metabolic processes, exhibited a similar trend of diminished levels in the ulcer group, highlighting disrupted regulatory mechanisms. Following treatment, particularly with OMP-NS, notable improvements in PPAR-γ levels were observed, indicating the potential of these interventions in restoring anti-inflammatory responses. Furthermore, SIRT-1 had decreased levels in the ulcer group, indicating impaired cellular stress responses. The administration of OMP and OMP-NS yielded divergent effects on SIRT-1 levels (Figure 12). In addition, the OMP-NS significantly upregulated Nrf2/PPAR-γ/SIRT-1 levels more efficiently than OMP, indicating its potential role in augmenting antioxidant pathways and restoring anti-inflammatory responses.

### 3.14. Impact of OMP and OMP-NS on Histological Alterations in Gastric Mucosa in Ethanol-Induced Ulceration

**Figure 13:**
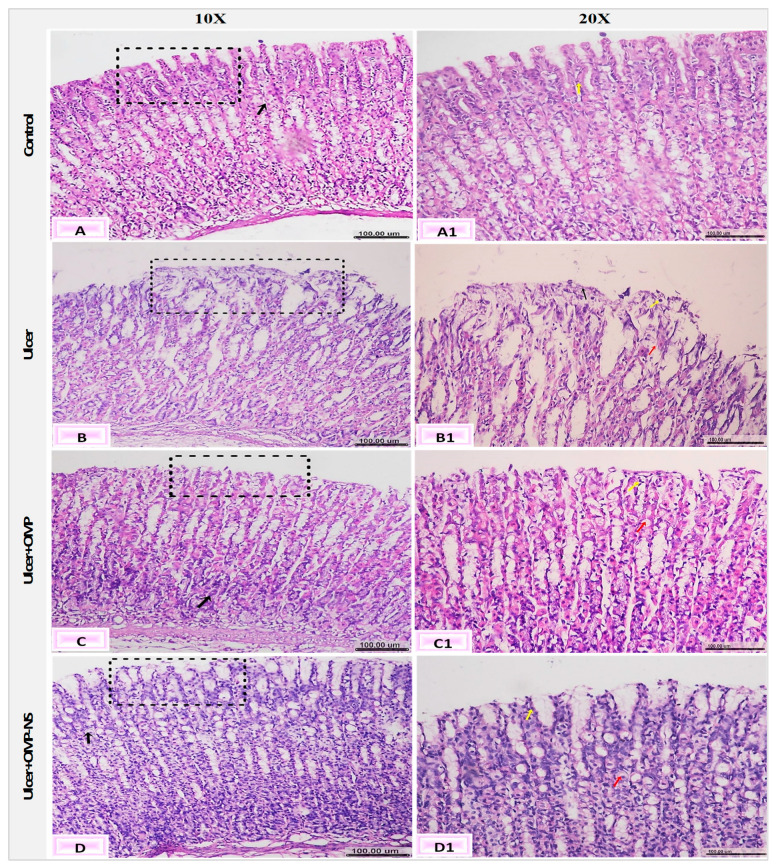
Photomicrographs of the gastric mucosa and submucosa layers showing (**A**) normal fundic mucosa (black square), intact lamina propria (black arrow), and (**A1**) gastric pits (yellow arrow); (**B**) erosions and ulcerative lesions (black square) and (**B1**) edema (red arrow) and cell debris (yellow arrow); (**C**) desquamation of the lining epithelium (black square), degeneration of gastric glands (black arrow), and (**C1**) pyknotic nuclei of the epithelial lining (yellow arrow) and vacuolar degeneration (red arrow); and (**D**) apparently healthy mucosa and submucosa layers (black square), vacuolation of parietal cells (black arrow), and (**D1**) mucous exudate (yellow ar-row), and vacuolar degeneration (red arrow) (H&E, scale bar: 100 µm).

The histological investigation of the fundic mucosa in the control group revealed intact surface epithelium, composed of simple columnar mucous-secreting cells that extend into the lamina propria, creating gastric pits (Figure 13A). Fundic glands extend perpendicularly to the surface, encompassing the entire thickness of the lamina propria (Figure 13A1). Conversely, the ulcer group exhibited gastric ulcers marked by severe histopathological changes in gastric tissue, including pronounced erosions and ulcerative lesions of the tunica mucosa, characterized by desquamation, sloughing of the fundic mucosa, and degradation of the basement membrane of the mucosal layer (Figure 13B). Furthermore, there exists a combination of cellular debris and infiltrating inflammatory cells (Figure 13B1). 

The ulcer + OMP group exhibited mild amelioration in the fundic mucosa, characterized by degeneration of the gastric glands, pyknotic nuclei in the epithelial lining, and vacuolar degeneration (Figure 13C,C1). However, the ulcer + OMP-NS group had a more pronounced enhancement in the histological architecture of gastric layers, where minor changes were observed as vacuolar degeneration of the epithelial lining of the tunica mucosa. Furthermore, the regeneration of the superficial layer was observed (Figure 13D,D1). 

**Figure 14:**
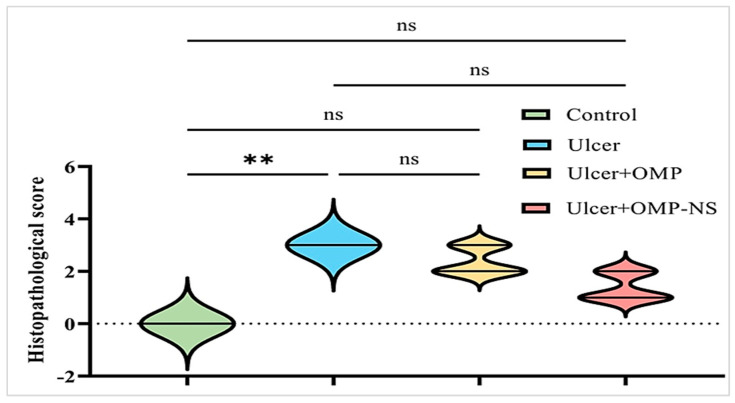
Scoring of histopathological alterations in each experimental group. All results were presented as median with interquartile range (IQR) by Kruskal–Wallis test. ns: non-significant, ** *p* < 0.01.

The scoring of histopathological alterations was recorded in Figure 14. The ulcer group exhibited a significant increase in histological damage scores compared to the control group (*p* < 0.05). Although both OMP and OMP-NS treatments showed tendencies for enhancement, the disparities in histology scores between the treated groups and the ulcer group were not statistically significant (*p* > 0.05). Nevertheless, qualitative microscopy indicated that OMP-NS treatment resulted in more pronounced histological enhancement than conventional OMP, particularly in the regeneration of epithelial layers and reduced inflammatory infiltration.

## 4. Discussion

Ethanol-induced ulcers are commonly used as a model to assess the efficacy of various drugs in ulcer treatment; it is administered orally to experimental animals to generate ulcers and gastrointestinal lesions [36]. OMP is extensively utilized for the treatment of gastrointestinal ulcers; however, it exhibits low solubility and is linked to numerous adverse effects. The aim of this study was to create and characterize OMP-NS with enhanced solubility and bioavailability and to assess its therapeutic effects on ethanol-induced gastric injury in rats, comparing its efficacy to traditional OMP therapy. 

The characterization of the OMP-NS was assessed using DLS, zeta potential, TEM, XRD, FTIR spectroscopy, UV spectrophotometry, in vitro release tests, and EE%, which provide essential confirmation of the nanoparticle formulation’s creation. The created OMP-NS demonstrated particle size and PDI values of 216.1 nm and 0.2, respectively, signifying a narrow particle size distribution. These values are similar to those published by Elshafeey and El-Dahmy [37], who indicated that the produced NS exhibited particle size and PDI values of 217.09 ± 4.18 nm and 0.46 ± 0.27, respectively. Singh et al. [38] suggest that a PDI under 0.3 is often preferable. Moreover, Leung [39] reported that NS are colloidal suspensions of drug nanocrystals, generally measuring between 100 and 400 nm in diameter.

Zeta potential is an essential measure for assessing the physical stability of formulated NS. The zeta potential value of the formulated OMP-NS was −19.6 mV. The negative zeta potential indicates sufficient electrostatic stabilization, essential for preventing agglomeration and ensuring the stability of the nanosuspension [40]. Furthermore, nanoparticles exhibiting an appropriate zeta potential impede aggregation by generating repulsive forces and augmenting permeability across cell membranes [41]. Due to the predominantly negative surface charge of plasma proteins, it has been shown that negatively charged nanoparticles circulate more favorably in blood than positively charged ones [42]. TEM images depicted the spherical particles of the generated nanoparticles [43], with a mean size of 67.28 nm. This reduction in size is crucial for enhancing the solubility and bioavailability of OMP, as smaller particles increase the surface area of the particles, facilitating enhanced contact with the solvent and promoting dissolution [44]. 

The FTIR analysis demonstrated notable shifts and the emergence of new peaks between OMP and its NS formulation; these spectral alterations suggest that the NS formulation may augment the solubility and stability of OMP. A similar finding published by Gera et al. [45] indicated that NS of poorly soluble medicines exhibited enhanced oral bioavailability. The XRD pattern of the OMP-NS exhibited a notable decline in peak intensity and a reduction in the number of observable peaks, indicating that some of the OMP has converted to an amorphous state. Amorphous drugs generally demonstrate significantly greater apparent solubility than their crystalline counterparts due to their elevated energy state, which facilitates the formation of a supersaturated condition in the gastrointestinal tract, hence enhancing bioavailability [46]. 

Furthermore, the UV spectra of the OMP-NS exhibited a shift in the absorbance peaks relative to the raw OMP, indicating improved solubility and dispersion of OMP in its NS form. The current analysis demonstrated an in vitro drug release of 77.33% after one hour, with a cumulative release of 97.78% at eight hours, indicating that the NS formulation improved the solubility and bioavailability of OMP. A quick drug release rate of OMP-NS can effectively alleviate pain from ulcer symptoms and enhance bioavailability, particularly for poorly soluble drugs. Additionally, our findings revealed that the OMP-NS achieved a high EE% of 96.97%. These results are comparable to those reported by Gupta et al. [21], which indicated that the OMP-loaded NS exhibited higher EE (89.09 ± 0.6%) and an in vitro drug release of 87.3% at 60 min.

The consumption of ethanol resulted in hemorrhagic mucosal injury and the production of ROS, consequently increasing the susceptibility of stomach mucosal layers to ulcerative injuries [47,48]. Our findings indicated that the oral administration of ethanol resulted in pronounced hemorrhagic lesions on the gastric mucosa, a considerable increase in stomach weight, the stomach coefficient, and the U.I., and reduced gastric pH values. Similar impacts of ethanol intake on the stomach mucosa were reported by Jabbar et al. [36] and Jabbar et al. [49]. The changes in stomach weight may indicate alterations in gastric motility, inflammation, or tissue damage, which are relevant factors concerning ethanol-induced ulceration [50]. Moreover, our observation of a reduced stomach pH aligns with the results of Obied [51], who demonstrated that acute ethanol intake elevated acid production, thereby reducing the pH of gastric juice. Conversely, both treatment groups showed significant efficacy in enhancing multiple parameters related to stomach health in cases of ethanol-induced gastric injury. In addition, our findings indicated that the OMP-NS exhibited an even higher effect in this regard. The prior research conducted by Wetchakul et al. [52] demonstrated that pretreatment of rats with OMP (20 mg/kg), used as a standard reference, markedly elevated pH levels. 

The results of the current investigation revealed substantial changes in enzyme levels related to liver and kidney function in the ulcer group. These findings align with previous studies by Ghareeb et al. [53], demonstrating that ethanol consumption resulted in liver and kidney impairment and damage. Conversely, our findings indicated that both OMP and OMP-NS considerably improved the biochemical changes linked to ethanol-induced gastric mucosal injury, as seen by enhancements in ALT, AST, ALP, creatinine, urea, and uric acid levels relative to the ulcer group. On the other hand, the results of the present investigation indicated that ethanol consumption affects cortisol and gastrin levels. In agreement with our findings, Sun et al. [54] demonstrated that gastrin levels were increased in the plasma of rats with gastric ulcers. Conversely, both OMP and OMP-NS treatments led to a decrease in cortisol and gastrin levels, and OMP-NS demonstrated an even greater improvement in mitigating these hormonal responses.

Ethanol-induced gastric ulcers are linked to the accumulation of inflammatory cells and elevated production of ROS, which may subsequently trigger oxidative damage [55,56]. The ROS interact with lipids to produce lipid peroxides, contributing to significant damage [57]. MDA is the end product of lipid peroxidation and is widely used as a reliable marker of lipid peroxidation [58]. The present study demonstrated that oral administration of ethanol induced considerable oxidative stress in the ulcer group by elevating levels of ROS and MDA while diminishing the synthesis of SOD in gastric tissue homogenates [3]. In contrast, rats administered OMP exhibited markedly elevated levels of SOD and reduced concentrations of MDA and ROS in gastric tissues [59]. Moreover, OMP-NS resulted in a more pronounced effect in reinstating antioxidant defenses while diminishing oxidative indicators linked to stomach ulcers. 

Normal NO levels play a protective role in the gastric mucosa [60]. The presence of gastric mucosal lesions significantly elevates the expression of inducible nitric oxide synthase (iNOS) [61], resulting in elevated NO synthesis that facilitates neutrophil infiltration and activation in the stomach mucosa and causes considerable damage [62]. The current findings indicated that the ulcer group exhibited a significant increase in NO levels, while treatment with OMP or OMP-NS induced a notable decrease in NO levels. The prior research conducted by Wetchakul et al. [52] revealed that OMP, serving as a reference drug, enhanced the decrease in iNOS levels in rats with ethanol-induced stomach ulcers.

In addition, the oxidative stress damage to the stomach mucosa generated by ethanol was associated with the overexpression of NADPH oxidases (NOXs) [63]. NOXs are a group of enzymes significantly implicated in oxidative stress [64]. In the current result, the ulcer group exhibited a dramatic increase in NADPH levels. This marked increase indicates heightened activity of NOX enzymes, which utilize NADPH to generate superoxide anions, leading to increased oxidative stress and inflammation in the ulcerative state. Consistent with our findings, Badr et al. [4] showed that the expression of NOX in gastric tissues was markedly elevated in the ethanol-ulcer group. In contrast, OMP or OMP-NS treatment resulted in a substantial decrease in NADPH levels. Additionally, treatment with OMP-NS produced even more pronounced effects in this regard. These findings indicated that OMP-NS not only reduces oxidative stress but also effectively modulates the NOX pathway, thereby minimizing the substrate (NADPH) necessary for ROS generation.

Inflammation is a significant factor linked to the increased formation of ROS associated with ethanol-induced gastric injury. HMGB1 is a proinflammatory mediator linked to the delayed healing of gastric ulcers. It attaches to many receptors, but when it binds to TLR4, it activates NF-κB and induces TNF-α [65,66]. NF-κB has been recognized as a modulator of gene regulation related to inflammatory responses in gastric epithelial cells, and it is connected with the IκB protein in the cytoplasm [67]. In response to several inflammatory stimuli, including ROS, NF-κB separates from IκB and migrates to the nucleus, where it activates the transcription of additional pro-inflammatory cytokines, such as IL-1β and TNF-α [68]. TNF-α promotes neutrophil infiltration into the gastric mucosa, triggering an inflammatory response that can impede microcirculation at the ulcer periphery, cell proliferation, and vascular regeneration, hence prolonging ulcer healing [4,69]. Moreover, higher levels of TNF-α may induce the release of IL-6 and other cytokines, hence amplifying the inflammatory response [70]. In addition, NLRP3 is an intracellular cytoplasmic multiprotein implicated in several inflammatory diseases [71]. Numerous studies have demonstrated that reducing NLRP3 activity relieves ethanol-induced gastrointestinal injury [72,73]. Consistent with these findings, ethanol in this investigation induced inflammation by significantly elevating HMGB1, which subsequently increased NF-κBp56, NLRP3, TNF-α, IL-1β, and IL-6 levels. In accordance with our findings, Shams and Eissa [74] demonstrated that ethanol induced inflammation by markedly increasing HMGB1, NF-κBp56, and TNF-α levels. Furthermore, our results aligned with those of Raish et al. [3], which indicated that oral administration of ethanol significantly elevated proinflammatory cytokines in ulcerative rats. 

Conversely, treatment with OMP or OMP-NS showed significant reductions in HMGB1, NLRP3, NF-κB, and proinflammatory cytokine levels, indicating potential in mitigating inflammation and promoting healing in gastric ulcers. Our findings corroborate earlier research indicating that OMP, used as a reference drug, significantly diminished the levels of HMGB1, NF-κB p65, and NLRP3 in comparison to the ulcer group [75] and substantially reduced the levels of IL-1β, IL-6, and TNF-α in gastric mucosa [76]. Additional observations revealed that OMP-NS treatments had shown more favorable outcomes than OMP in this regard; this may be attributed to the improved bioavailability, better absorption, and sustained release characteristics of the nanosuspension formulation. The enhanced effectiveness of OMP-NS suggests its promising therapeutic potential for modulating inflammatory responses and facilitating healing in gastric ulcers.

TLR-2 is a key part of the immune response in the gastrointestinal mucosa and can induce inflammation upon activation. It is recognized as a significant contributor to inflammatory reactions [77]. MyD88 serves as the main adapter for TLR-2. Previous research indicated that the activation of the TLR-2/MyD88 signaling pathway is crucial in the development and progression of gastrointestinal tract diseases [78]. Our findings indicated significant elevations in TLR-2/MyD88 levels in the ulcer group. These findings underscored the pivotal role of the TLR-2 MyD88 pathway in the inflammatory cascade associated with gastric ulcers. In contrast, treatment with OMP or OMP-NS had a significant ability to modify TLR-2/MyD88. In line with our results, Fu et al. [79] indicated that OMP (used as a reference drug) markedly reduced TLR-2/MyD88 co-localization in the stomach mucosa relative to rats with gastric ulcers. Furthermore, treatment with OMP-NS produced even more pronounced effects in this regard.

On the other hand, several factors mediate their gastroprotection against oxidative damage by mitigating the inflammatory response, including Nrf2, PPAR-γ, and SIRT-1. The Nrf2 signaling pathway is crucial for preserving cells against oxidative damage, especially in the gastrointestinal system. It mitigates inflammation by downregulating NF-κB and subsequent proinflammatory signaling [8]. Consistent with prior research, our findings indicated that the ulcer group had a notable reduction in Nrf2 [80,81]. Notably, Nrf2 plays an essential role in regulating the activity of HMGB1 [82,83], and a previous study on ethanol-induced gastric ulcers demonstrated a negative correlation between HMGB1 and Nrf2 levels [4]. 

SIRT1 is another critical component in gastroprotection, since it regulates the gene expression of several proteins involved in the pathogenesis of multiple diseases and toxic injuries [84,85]. SIRT can augment Nrf2 synthesis, known for activating antioxidant proteins like SOD [86], while downregulating NF-κB protein expression, the primary activator of the inflammatory response [87,88]. Furthermore, ethanol-induced gastrointestinal injury is associated with a significant decline in the production of the cytoprotective protein PPAR-γ [89]. PPAR-γ regulates the secretion of inflammatory cytokines from injured tissue and immune cells, including TNF-α and IL-β [90]. It modulates the inflammatory response generated by oxidative stress through the upregulation of antioxidant enzyme expression [91]. Gupta et al. [92] demonstrated that PPAR-γ agonists suppress NF-κB activation and cellular proliferation in gastrointestinal cells. Moreover, it protects the stomach and promotes ulcer healing in numerous experimental models of gastric ulcers [93,94]. Consistent with these findings, our results indicated that the ulcer group had a notable reduction in the expression of SIRT and PPAR-γ. In contrast, treatment with OMP or OMP-NS markedly elevated the expression of Nrf2, PPAR-γ, and SIRT-1, suggesting a potential function for these treatments in augmenting antioxidant pathways and reinstating anti-inflammatory responses. In accordance with our findings, a previous study indicated that rats administered OMP used as a reference drug displayed elevated Nrf2 expression in ethanol-induced stomach ulcers [53]. Similarly, El-Shitany et al. [95] indicated that OMP, used as a reference drug, contributed to significant increases in gastric PPAR-γ mRNA expression compared to the ulcer group. 

Eventually, macroscopic inspection and histological analysis in this study confirmed the gastric injury induced by ethanol administration; these results are consistent with previous studies [3,96,97]. Although OMP and OMP-NS showed histological improvements, no significant differences were observed in the histological scores compared to the ulcer group. This lack of histological improvement may be attributed to the limited treatment time, which might not be sufficient to allow for substantial tissue regeneration and structural recovery.

Overall, OMP-NS treatments demonstrated enhanced efficacy outcomes relative to OMP treatment responses for gastric ulcers, as evidenced by their impact on the various markers identified in this study. This effect can be attributed to enhanced absorption and increased drug concentration at the site of action, resulting in more effective activation of antioxidant pathways and anti-inflammatory responses. Moreover, the sustained release profile of OMP-NS may provide a continuous therapeutic effect, promoting prolonged activation of Nrf2, PPAR-γ, and SIRT-1, which are crucial for the healing of gastric ulcers. Nonetheless, despite these beneficial outcomes, histological examination indicated that neither OMP nor OMP-NS treatment resulted in improvements in histological scores relative to the ulcer group, further underscoring the need for extended treatment durations in future studies.

### Strengths and Limitations of the Study

This study possesses numerous notable strengths: first, the creation of OMP-NS to improve solubility and therapeutic efficacy, given the known limitations of traditional OMP. The formulation underwent comprehensive analysis using various techniques. Furthermore, the study integrated biochemical, molecular, and histological evaluations to examine the therapeutic effects, providing a profound understanding of their mechanism of action. Nevertheless, the study has some limitations. An important limitation is the limited number of animals per group, which may reduce statistical power and restrict the generalizability of the results. Additionally, although only male rats were included to reduce variability, this limits the applicability of the findings to both sexes. Including female subjects in future studies would help determine whether sex-based differences affect treatment outcomes. Another limitation is the short treatment and monitoring duration of seven days. Although OMP-NS demonstrated histological enhancements, the histological scores did not attain statistical significance relative to the ulcer group. These issues may result from the limited duration, which may not have permitted adequate time for complete histological recovery. Prolonging the treatment duration may result in enhanced tissue regeneration and statistically significant histopathological outcomes.

## 5. Conclusions

The findings of this study demonstrated that the formulation of OMP-NS significantly enhances the therapeutic potential of OMP in treating ethanol-induced gastric ulcers in male rats. The characterization of OMP-NS revealed favorable physicochemical properties, including a small particle size and high entrapment efficiency, which contribute to improved solubility and bioavailability. In vivo experiments indicated that OMP-NS effectively reduced oxidative stress markers and inflammatory responses associated with gastric injury, outperforming traditional OMP therapy. The observed upregulation of protective pathways, including Nrf2/PPAR-γ/SIRT1, further supports the notion that OMP-NS not only alleviates gastric damage but also promotes healing through antioxidant and anti-inflammatory mechanisms.

## Figures and Tables

**Table 1:** Primer’s sequence of all studied genes.

	Forward Sequence	Reverse Sequence
PPAR-γ	GTACTGCCGCTTCCAGAA	GTGCACGCCATACTTGAG
SIRT-1	TGTTTCCTGTGGGATACCTGA	TGAAGAATGGTCTTGGGTCTTT
Nrf2	CACATCCAGACAGACACCAGT	CTACAAATGGGAATGTCTCTGC
GAPDH	TGGATTTGGACGCATTGGTC	TTTGCACTGGTACGTGTTGAT

PPAR-γ: peroxisome proliferator-activated receptor gamma; SIRT-1: sirtuin 1; Nrf2: nuclear factor erythroid 2-related factor 2; GAPDH: glyceraldehyde-3-phosphate dehydrogenase.

**Table 2:** Characteristic FTIR peaks of OMP and OMP-NS.

NO	Functional Group	Frequency Range	Observed Frequency OMP	Observed Frequency OMP-NS
1	C=C aromatic	1600–1400 cm^−1^	1428.05 cm^−1^	1418.18 cm^−1^
2	C-OH stretch	1200–1020 cm^−1^	1066.97 cm^−1^	1010.93 cm^−1^
5	C-Br	750–500 cm^−1^	548.34 cm^−1^	668.57 cm^−1^
6	C-I	~500 cm^−1^	471.98 cm^−1^	448.62 cm^−1^

**Table 3:** Effects of treatments on body weight, stomach weight, stomach coefficient, gastric pH, U.I., and CR.

	Control	Ulcer	Ulcer + OMP	Ulcer + OMP-NS
Final body weight (g)	255.7 ± 10.05	186 ± 11.2 ^@^	213 ± 9.4 ^@#^	235.5 ± 5.9 ^@#&^
Stomach weight (g)	1.17 ± 0.1	1.85 ± 0.07 ^@^	1.29± 0.20 ^#^	1.06 ± 0.12 ^#^
Stomach coefficient (%)	0.46 ± 0.05	0.99 ± 0.08 ^@^	0.61 ± 0.06 ^@#^	0.45 ± 0.04 ^#&^
Gastric PH	6.28 ± 0.22	4.17 ± 0.25 ^@^	4.34 ± 0.31 ^@^	5.39 ± 0.45 ^@#&^
UI (%)	0.0 ± 0.0	6.05 ± 0.51 ^@^	1.37 ± 0.27 ^@#^	0.55 ± 0.14 ^@#&^
CR (%)	**-**	**-**	77.19 ± 5.20 ^@#^	90.83 ± 2.65 ^@#&^

Data were expressed as mean ± SD. ^@^ Significant vs. the control group, ^#^ Significant vs. the ulcer group, ^&^ Significant between ulcer + OMP and ulcer + OMP-NS groups at *p* < 0.05.

**Table 4:** Markers of liver and kidney function in ethanol-induced gastric mucosal damage.

	Control	Ulcer	Ulcer + OMP	Ulcer + OMP-NS
ALT (U/mL)	33.48 ± 1.03	91.97 ± 2.68 ^@^	69.27 ± 2.31 ^@#^	48.27 ± 3.42 ^@#&^
AST (U/mL)	44.90 ± 1.35	113 ± 1.26 ^@^	80.64 ± 3.05 ^@#^	60.06 ± 1.79 ^@#&^
ALP (ng/mL)	2.04 ± 0.09	8.65 ± 0.06 ^@^	6.01 ± 0.03 ^@#^	4.01 ± 0.9 ^@#&^
Creatinine (mg/dL)	2.45 ± 0.09	13.42 ± 0.57 ^@^	7.48 ± 0.37 ^@#^	5.07 ± 0.74 ^@#&^
Urea (nmol/mL)	1.77 ± 0.03	9.48 ± 0.37 ^@^	6.33 ± 0.08 ^@#^	3.92 ± 0.07 ^@#&^
Uric acid (mg/dL)	0.50 ± 0.03	5.48 ± 0.37 ^@^	3.28 ± 0.29 ^@#^	1.75 ± 0.09 ^@#&^

Data were expressed as mean ± SD. ^@^ Significant vs. the control group, ^#^ Significant vs. the ulcer group, ^&^ Significant between ulcer + OMP and ulcer + OMP-NS groups at *p* < 0.05.

## Data Availability

The original contributions presented in this study are included in the article. Further inquiries can be directed to the corresponding authors.
